# Finished Whole-Genome Sequences of Two *Clostridium botulinum* Type A(B) Isolates

**DOI:** 10.1128/genomeA.00381-17

**Published:** 2017-05-25

**Authors:** Jessica L. Halpin, Karen Hill, Shannon L. Johnson, David Carlton Bruce, T. Brian Shirey, Janet K. Dykes, Carolina Lúquez

**Affiliations:** a Centers for Disease Control and Prevention, Atlanta, Georgia, USA; b Los Alamos National Laboratory, Los Alamos, New Mexico, USA

## Abstract

*Clostridium botulinum* secretes a potent neurotoxin that causes devastating effects when ingested, including paralysis and death if not treated. In the United States, some clinically significant strains produce toxin type A while also harboring a silent B gene. These are the first two closed genome sequences published for this subset.

## GENOME ANNOUNCEMENT

Many *Clostridium botulinum* type A strains isolated in the United States also harbor a silent toxin type B gene (*bont*/B) (our unpublished data). These strains have been difficult to distinguish by traditional subtyping methods, such as pulsed-field gel electrophoresis or multilocus variable-number tandem-repeat analysis (1–8). As whole-genome sequencing has become more accessible to public health laboratories, it is vital to have representative closed genome sequences for clinically relevant strain populations. Here, we present two finished genome sequences containing a *bont*/A1 gene and the unexpressed *bont*/B5 gene.

A hybrid assembly method was utilized, and both isolates were sequenced by Illumina and PacBio and then assembled using Velvet 1.2.08, HGAP 3, and Phrap 4.24. The coverages for strain CDC69094 were 263× by PacBio and 309× by Illumina. The coverages for strain CDC67190 were 334× by PacBio and 310× by Illumina.

CDC67190 was isolated from patient stool in the course of a foodborne outbreak investigation. The finished genome included 2 plasmids, pNPD12_1 (accession no. CP014146), with 5,926 bp, and pNPD12_2 (accession no. CP014147), with 59,360 bp. These plasmids contain 6 and 109 coding regions, respectively. The chromosome has 3,954,777 bp and includes 3,602 coding regions and a G+C content of 28.2%. Both toxin genes were found in the chromosome rather than on the plasmids.

CDC69094 was isolated from a stool specimen from an infant botulism case. Interestingly, this genome did not include a plasmid, indicating that the unexpressed B gene is contained on the chromosome with the toxin type A gene. The chromosome (accession no. CP013246) included 4,089,027 bp, a G+C content of 28.1%, and 3,711 coding regions.

### Accession number(s).

The finished sequences have been deposited in GenBank, under accession no. CP013246 for CDC69094 and accession numbers CP014146, CP014147, and CP014148 for CDC67190.
